# Identification and Validation of Novel Serum Autoantibody Biomarkers for Early Detection of Colorectal Cancer and Advanced Adenoma

**DOI:** 10.3389/fonc.2020.01081

**Published:** 2020-07-22

**Authors:** Hejing Wang, Bei Zhang, Xiaojin Li, Donghu Zhou, Yanmeng Li, Siyu Jia, Saiping Qi, Anjian Xu, Xiaomu Zhao, Jin Wang, Zhigang Bai, Bangwei Cao, Ni Li, Min Dai, Hongda Chen, Jian Huang

**Affiliations:** ^1^Experimental Centre, Beijing Friendship Hospital, Capital Medical University, Beijing, China; ^2^Department of General Surgery, Beijing Friendship Hospital, Capital Medical University, Beijing, China; ^3^National Clinical Research Center for Digestive Disease, Beijing Friendship Hospital, Capital Medical University, Beijing, China; ^4^Department of Oncology, Beijing Friendship Hospital, Capital Medical University, Beijing, China; ^5^Office of Cancer Screening, National Cancer Centre/National Clinical Research Centre for Cancer/Cancer Hospital, Chinese Academy of Medical Sciences and Peking Union Medical College, Beijing, China

**Keywords:** colorectal cancer, advanced adenoma, biomarker, tumor-associated antigen, ALDH1B1

## Abstract

**Background:** Colorectal cancer (CRC) comprises a large proportion of malignant tumors, and early detection of CRC is critical for effective treatment and optimal prognosis. We aimed to discover and validate serum autoantibodies for early detection of CRC.

**Methods:** Combined with CRC-associated autoantibodies discovered by serological proteome and multiplex analyses, 26 predefined autoantibodies were evaluated in 315 samples (130 CRCs, 75 advanced adenomas, and 110 healthy controls) by protein microarray analysis. Autoantibodies with potential detection value were verified by enzyme-linked immunosorbent assays (ELISAs). Receiver operating characteristic (ROC) curve analysis was conducted to evaluate the accuracy of the biomarkers.

**Results:** Four serum autoantibodies (ALDH1B1, UQCRC1, CTAG1, and CENPF) showed statistically different levels between patients with advanced neoplasm (CRC or advanced adenoma) and controls in microarray analysis, which were validated by ELISAs. Among the four biomarkers, the ALDH1B1 autoantibody showed the highest detection value with area under the curve (AUC) values of 0.70 and 0.74 to detect CRC and advanced adenoma with sensitivities of 75.68 and 62.31% and specificities of 63.06 and 73.87%, respectively. By combining the four biomarkers, the performance was improved with an AUC of 0.79 to detect CRC and advanced adenomas.

**Conclusion:** The ALDH1B1 autoantibody has a good potential for early detection of CRC and advanced adenoma, and measuring serum autoantibodies against tumor-associated antigens may improve detection of early CRC.

## Introduction

Cancer is one of the leading causes of death in the developed world. It is estimated that 18.1 million new cancer cases and 9.6 million cancer deaths occurred in 2018, with colorectal cancer (CRC) accounting for 1.8 million new cases and 881,000 deaths (1). The cancer death rate in the USA decreased by 26% from its peak of 215.1 deaths per 100,000 people in 1991 to 158.6 deaths in 2015. Specifically, the death rate of CRC was reduced to 52% between 1970 and 2015 (2). This reduction in mortality is attributed to improvements in treatment (12%), changing patterns in CRC risk factors (35%), and screening uptake (53%) (3). Stool-based tests (faecalis immunochemical, faecalis occult blood, and multi-targeted stool DNA tests) and tests that directly visualize the colon (colonoscopy, sigmoidoscopy, and computed tomographic colonography) are recommended for CRC screening. However, the CRC screening rate in 2016 for federally qualified health centers in the USA was 39.9%. At least 40% of age-eligible adults do not adhere to screening guidelines (4). Faucal immunochemical and occult blood tests are advised, but the 70% sensitivity must be improved (5). Molecular screening methods examining abnormal protein and mRNA expression, and gene mutations, are conducted on a large scale. In October 2014, a non-invasive stool-based DNA test from Exact Sciences (Cologuard) for average-risk patients was the first Food and Drug Administration-approved test with sensitivity of 92% and specificity of 87% (6–8). However, for advanced precancerous lesions, the sensitivities of DNA and faecalis immunochemical tests were 42.4% and only 23.8%, respectively (8). A systematic review disclosed overall sensitivities for CRC detection by fecal DNA markers ranging from 53 to 87% with varying specificities above 76%. A study using MALDI-TOF MS combined with magnetic beads identified a panel of serum protein biomarkers, which showed sensitivity and specificity above 85% for all stages of CRC (9), implying the potential value of blood tests of protein markers to detect CRC. Although there have been a series of studies on tumor biomarkers in CRC, the value in clinical application is very limited. Further evaluation of their detection value in CRC and identification of novel serum biomarkers are crucial for early detection of CRC.

Starting in the 1950s, studies focused on relationships between autoantibodies and cancer. Autoantibodies were assessed as biomarkers for cancer diagnosis because their production may precede clinical confirmation of a tumor by several months to years. A study had demonstrated that an anti-p53 autoantibody was significantly correlated with subsequent development of malignancy with a predictive value of 0.76 and average lead time to diagnosis of 3.5 years (10). Tumor-associated autoantibodies (TAAbs) have been found during the transition to malignancy (11). Autoantibodies can stably exist at high levels induced by immune cascades despite low levels of the corresponding antigen. Therefore, autoantibodies have been proposed as biomarkers for early-stage detection of cancers. In recent years, autoantibodies against TAAs have been reported in patients with CRC, but only by a series of studies on small cohorts and without high-throughput screening in a large cohort of samples especially advanced adenoma (AA), an advanced precancerous lesion (12, 13).

In our study, we identified TAAbs to improve early detection of CRC. We employed serological proteome analysis (SERPA) to initially screen total proteins from CRC tissues to identify discrepant autoantibodies of TAAs between healthy persons and CRC patients. Subsequently, protein microarrays and enzyme-linked immunosorbent assays (ELISAs) were used to further screen and confirm their value in diagnosing CRC and AA. We analyzed the value of single autoantibodies and panels of multiple autoantibodies against TAAs using receiver operating characteristics (ROCs) to find valuable biomarkers of early-stage CRC.

## Materials and Methods

### Study Population

The study was performed using a population-based CRC screening program conducted in 16 provinces of China from 2012 to 2016. The detailed study design has been described in a previous study (14). Briefly, all participants aged 40–69 years were first invited to undertake cancer risk assessment using an established risk score system. For participants who were evaluated as high risk, subsequent colonoscopy examination was conducted in designated hospitals. All eligible participants were invited to donate blood samples before the colonoscopy. The samples were handled according to SOPs and stored immediately at −80°C until use. In the current study, we recruited 315 participants including 130 CRC patients, 75 advanced adenoma patients, and 110 controls without colorectal neoplasms (Table S1). Advanced adenoma was defined as any adenoma measuring 10 mm or more, containing a substantial (>25%) villous component or exhibiting high-grade dysplasia. Final clinical diagnoses were classified according to the most advanced findings reported in the colonoscopy and/or histology report. Serum levels of carcinoembryonic antigen (CEA) were obtained from clinical records for CRC patients. In addition, six tumor tissue specimens and corresponding sera confirmed as CRC by pathological examination were obtained from the Department of General Surgery, Beijing Friendship Hospital, Capital Medical University. Correspondingly, serum samples from six healthy donors used in SERPA were obtained from the Health Examine Centre, Beijing Friendship Hospital, Capital Medical University.

The study was approved by the Ethics Committee of the Beijing Friendship Hospital, Capital Medical University, and the National Cancer Center/Cancer Hospital, Chinese Academy of Medical Sciences. All participants provided written informed consent.

### Blood Sample Preparation

Blood samples were collected by BD Vacutainer (product number: 367986) before colonoscopy in the designated hospitals by the study nurses. After completion of blood clotting, the blood samples were centrifuged at 1,200 g at room temperature for 12 min and the serum were aliquoted and stored at −80°C in the hospitals of the field provinces. All samples were then shipped to the central laboratory (National Cancer Center of China) through cold chain and stored in the freezer (−80°C) until further analyses.

### SERPA Analysis to Screen CRC-Associated Autoantibodies

Total protein from tumor tissues was extracted as described previously (15). Briefly, frozen tissue was lysed in 5 μl lysis buffer {7 mol/L urea, 2 mol/L thiourea, 4% 3-[(3-cholamidopropyl)-dimethylamino]-1-propanesulfonate, 1% dithiothreitol, 2% immobilized pH gradient (IPG) buffer (pH 3–10), and a protease inhibitor cocktail} per mg tissue and clarified by centrifugation. Protein concentrations were measured by a 2-D Quant Kit (GE Healthcare, Illinois, USA). Proteins were purified with a 2-D Clean-Up Kit (GE Healthcare). First, isoelectric focusing was performed on precast 18-cm immobilized pH 3–10 IPG strips (GE Healthcare) with 800 μg protein. Subsequently, sodium dodecyl sulfate polyacrylamide gel electrophoresis (SDS-PAGE) was performed and the proteins were transferred onto a polyvinylidene fluoride (PVDF) membrane, followed by incubation with serum samples from CRC patients or healthy controls as primary antibodies at a 1:100 dilution. Then, horseradish peroxidase (HRP)-conjugated goat anti-human IgG (Invitrogen, California, USA) was used as a secondary antibody at a 1:5,000 dilution. Immunoreactive spots on the PVDF membrane were detected by an enhanced chemiluminescence kit (Millipore, MA, USA), according to the manufacturer's instructions (15). The proteomic profile of proteins from the CRC tissue was used as a reference map for spot analysis. Spots on immunoblotting maps were matched to the reference map, and those detected in CRC serum, but not in healthy controls, were excised and identified by matrix-assisted laser desorption/ionization-time-of-flight (MALDI-TOF) mass spectrometry (Bruker Daltonics, Bremen, Germany). Mascot (Matrix Science, London, UK) was used for protein identification by searching the peak lists against the International Protein Index human database (15).

### Selection and Preparation of Tumor-Associated Antigens

In terms of the selection of TAA proteins for protein microarray analysis, we used 26 TAAs obtained from four sources: (1) SERPA identified five autoantibodies, CSRP1, SELENBP1, ALDH1B1, UQCRC1, and ENO1. (2) Our previous study evaluated 64 serum autoantibodies in CRC measured by multiplex bead-based serological assays (16). Autoantibodies against TP53, IMPDH2, MAGEA4, and MDM2 with underlying value, but without verification, were examined further in this study. (3) Autoantibodies against HSP60, CENPF, RGN, PRDX3, ACY1, ANXA4, and HINT1 that have been studied in hepatocellular carcinoma (15) and showed discrepancies in CRC in our preliminary experiments. (4) Autoantibodies against RPL13, RPH3AL, HMGN3, MPHOSPH6, IGF2BP1, VIL1, AIF1, CALR, CTAG1, and MYH13 based on previous systematic reviews of autoantibodies in CRC (12, 13).

Fragments or full-length recombinant proteins of selected TAAs, which were available for enzyme-linked immunosorbent assays (ELISAs), were purchased whenever possible. Recombinant CTAG1 and MYH13 proteins were prepared in-house.

### Protein Microarray Analysis for Detection of Autoantibodies

Preparation of the protein microarray and microarray detection of serum samples was performed according to our previous study (15). Briefly, the screened TAAs were robotically spotted in ordered arrays on aldehyde-activated glass slides by the microchip-spotting instrument PersonalArrayer™ 16 (CapitalBio, Beijing, China). Human IgG (Sigma, MO, USA) was spotted by a gradient and used as a positive control and for calibration to adjust the signal intensity of each small compartment, whereas a spot sample of phosphate-buffered saline (PBS) with 0.02% SDS and 1% glycerol, and PBS were used as negative controls. The serum to be tested was diluted at 1:10. Cy3-conjugated Donkey Anti-Human IgG (Sangon Biotech, Shanghai, China) diluted at 1:300 was used as a secondary antibody. The signals were collected using a GenePix® 4000B Microarray Scanner (Molecular Devices, California, USA) and analyzed by array vision 7.0 (Imaging Research, Catharines, Canada) (15). Receiver operating characteristic (ROC) curves to evaluate the performance of the biomarkers were plotted based on logistic regression models. The cutoff values for positive and negative reactivities were determined by the Youden index (15).

### Enzyme-Linked Immunosorbent Assays

Antigenic proteins ALDH1B1 (0.5 μg/ml), UQCRC1 (0.25 μg/ml), CTAG1 (8 μg/ml), and CENPF (2.0 μg/ml) were incubated in 96-well-microplates (Nunc A/S, Roskilde, Denmark) with 100 μl coating buffer (0.05 M carbonate/bicarbonate, pH 9.6) in each well. After incubation at 4°C overnight, free binding sites were blocked with 1% bovine serum albumin (BSA, Sigma) for ALDH1B1 and CTAG1, 1% casein (Sigma) for UQCRC1, and 10% newborn bovine serum (NBS, Gibco BRL, NY, USA) for CENPF. Standard serum was diluted at 1:40 (ALDH1B1, 1% BSA), 1:20 (UQCRC1, 10% NBS), 1:20 (CTAG1, 1% BSA), and 1:10 (CENPF, 10% NBS). A rabbit anti-human IgG-peroxidase antibody (Sigma) was added at 1:10,000, 1:15,000, 1:20,000, and 1:8,000. TMB HRP Substrate (Beijing Solarbio Science & Technology, Beijing, China) was reacted for 15 min at 37°C, which was stopped by addition of the stop solution (Beijing Solarbio Science & Technology). The absorbance was immediately read at 450 and 630 nm using a microplate reader (SpectraMax M3, Molecular Devices, CA, USA). The cutoff values for positive and negative reactivities were determined by the Youden index.

### Preparation of Recombinant ALDH1B1 Protein and Verification of the ALDH1B1 Autoantibody in Sera by Western Blotting

The whole coding sequence of ALDH1B1 with His tags was chemically synthesized and inserted into the prokaryotic expression plasmid pET-28a. The recombinant plasmid was transformed into T7 Express competent *E. coli*. Expression was induced by incubation with 0.1 M IPTG at 16°C for 4 h. After ultrasonication, dissolution of inclusion body proteins, and renaturation in dialysate, the expressed recombinant protein was purified by affinity chromatography using His Sefnose resin and analyzed by SDS-PAGE and Gel-Pro Analyzer version 3.1.00.00 as described above.

Then, the presence of autoantibodies against ALDH1B1 was verified by a western blot assay. Briefly, recombinant ALDH1B1 was electrophoresed on a 10% SDS-PAGE gel and then transferred onto a PVDF membrane that was blocked in 5% non-fat dry milk for 2 h at 37°C. The membrane was then cut into strips that were incubated separately with individual serum samples (1:500 dilution) and an anti-ALDH1B1 antibody (1:1,000; Abcam, Cambridge, UK) at 4°C overnight. The respective serum samples were mixed randomly, including those from healthy controls and patients with CRC and AA. The strips were incubated with horseradish peroxidase-conjugated anti-human IgG (1:5,000 dilution, Sigma) for 1 h at 37°C. The reactions were developed using enhanced chemiluminescence western blotting detection reagent (Millipore).

### Statistical Analysis

The chi-squared test or Student's *t*-test was used to determine the statistical significance between two groups. Receiver operating characteristic (ROC) curves to evaluate the performance of the markers were plotted based on logistic regression models. The cutoff was determined using a ROC curve and the Youden index. To overcome potential overfitting, we applied bootstrapping methods (1000 bootstrap samples) to adjust the areas under the curves and calculated the respective 95% confidence intervals (CIs). SPSS 22.0 for Windows (IBM, Chicago, IL, USA) was used for all statistical analyses, and *p* < 0.05 was considered as statistically significant.

## Results

### CRC-Related Autoantibodies Found by SERPA

Using an antigen library of a mixture of total proteins extracted from tumor tissues of six CRC cases, SERPA analysis was performed to screen CRC-related TAAs. Mixtures of serum samples from CRC cases and healthy controls were used as primary antibodies for western blot analyses. Figure S1A shows a representative Coomassie blue-stained 2-DE gel. Different patterns of reactivity were obtained by probing with CRC and normal control sera. Representative immunoreactive patterns with CRC and normal control sera are shown in Figures S1B,C. By comparing and matching the antigenic protein profiles of each 2-D immunoblot on the original 2-DE, protein spots that were frequently recognized by CRC serum, but not serum from normal controls, were excised from the gels and subjected to MALDI-TOF-MS analysis. Five TAAs were identified, which were associated with CRC (17–21), including autoantibodies against CSRP1, SELENBP1, ALDH1B1, UQCRC1, and ENO1, and applied to further evaluation by protein microarray. Information of the five candidate TAAs is presented in Table S2.

### Preparation of the Protein Microarray and Cohort Results Associated With TAAbs Identified by the Protein Microarray

**Figure 1:**
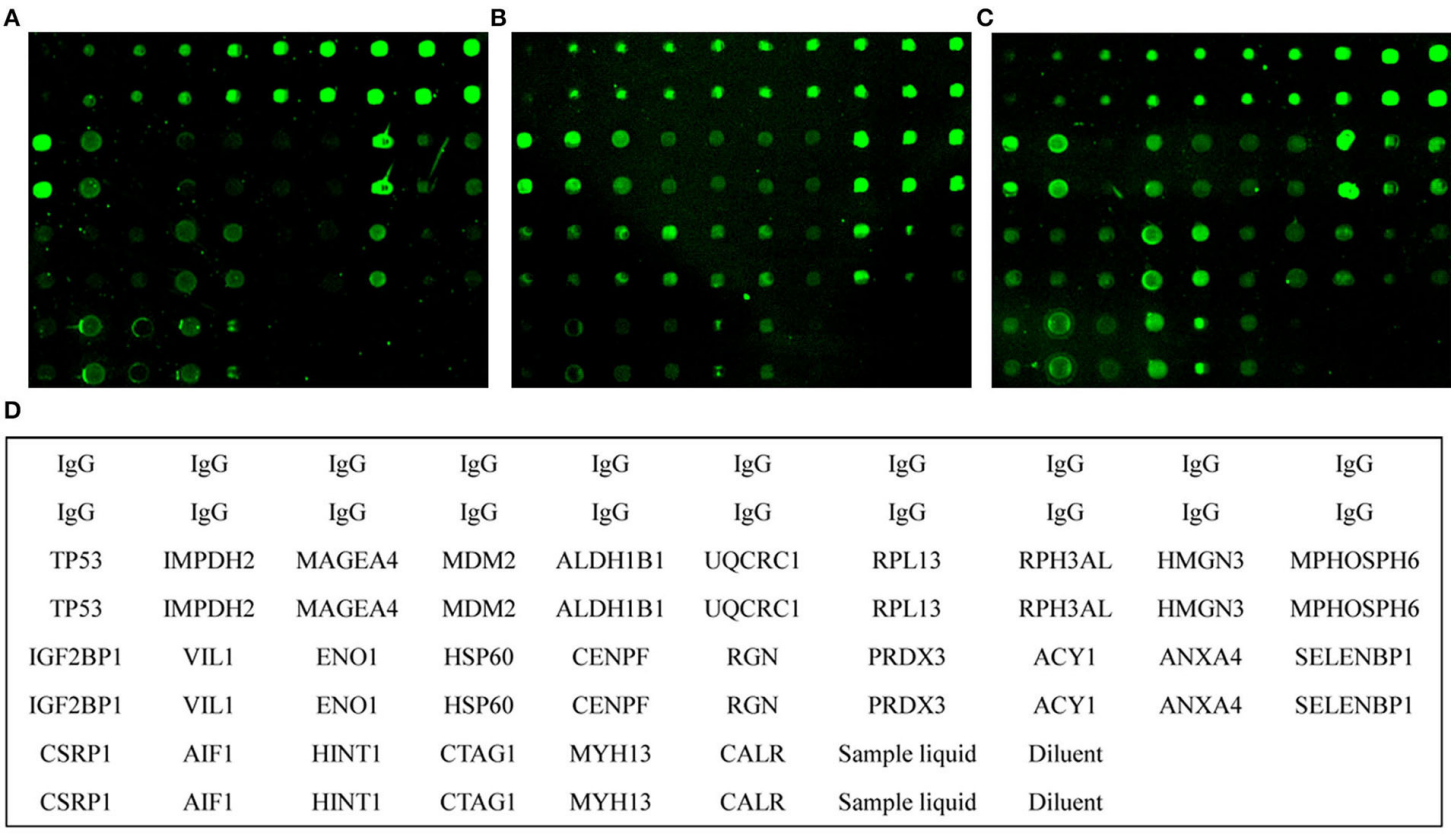
Microarray analysis of serum samples. Individual arrays were reacted with healthy control **(A)**, advanced adenoma **(B)**, and colorectal cancer **(C)** samples. **(D)**, Schematic diagram of individual proteins in the protein microarray matrix. Sample liquid, phosphate-buffered saline (PBS) with 0.02% SDS and 1% glycerol. Diluent, PBS.

To prepare the protein microarray, we used 26 antigenic proteins (Figure 1, Table S3). Twenty-four TAAs were purchased commercially, and two recombinant proteins, CTAG1 and MYH13, were prepared in-house (data not shown).

Using the protein microarray, the 26 antigens were detected simultaneously in 315 samples including 130 CRC, 75 AA, and 110 healthy control samples (Figure 1). For autoantibodies screened by SERPA, the results showed that autoantibodies against ALDH1B1 and UQCR1 may have underlying value for CRC patients with AUC values of 0.62 and 0.64, sensitivities of 37.60 and 47.20%, and specificities of 84.68 and 64.86%, respectively. However, CSRP1, SELENBP1, and ENO1 showed less value to detect CRC/AA with AUC values lower than 0.6 and a *P*-value around the threshold (Table S4). The results also showed that autoantibodies against TP53 may have a detection value for AA/CRC patients with AUC values of 0.65 and 0.67, sensitivities of 75.36 and 48.8%, and specificities of 55.86 and 76.58%, respectively. Furthermore, autoantibodies against CENPF and CTAG1 may have underlying detection values for AA with AUC values of 0.64 and 0.59, sensitivities of 59.42 and 57.97%, and specificities of 69.37 and 64.86%, respectively. In addition, other autoantibodies showed some potential detection value for CRC, including autoantibodies against IMPDH2, MDM2, HSP60, RPL13, VIL1, CENPF, RGN, and PRDX3. Detailed information about the various autoantibodies is presented in Table S4.

### Detection Value of TAAbs Reexamined by ELISAs

To confirm the detection value of the *TAAbs* for AA and CRC, we selected autoantibodies against ALDH1B1, UQCRC1, CTAG1, and CENPF for ELISAs based on the protein microarray results showing potential detection values for CRC or AA. Autoantibodies against ALDH1B1 and UQCRC1 screened by SERPA were first identified in CRC. The autoantibodies against CENPF have shown high specificity to detect AA. Autoantibodies against CTAG1 have recently been reported to show promising detection values for CRC and can be used as a reference for comparisons with other studies.

**Figure 2:**
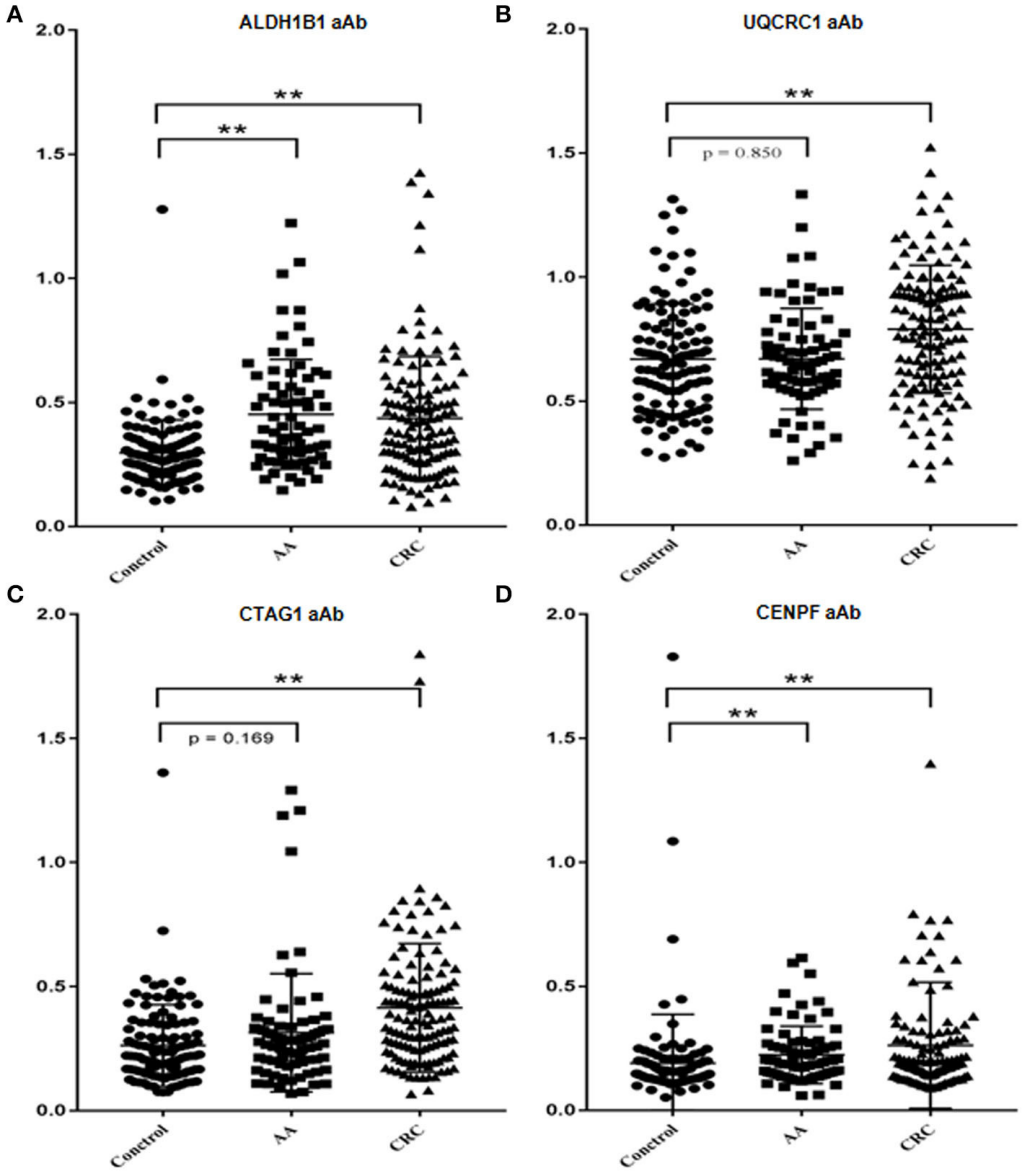
Comparison of the levels of autoantibodies against selected TAAs examined by ELISAs. Scatter diagram of the OD values of ALDH1B1 **(A)**, UQCRC1 **(B)**, CTAG1 **(C)**, and CENPF **(D)**. **p < 0.001.

**Figure 3:**
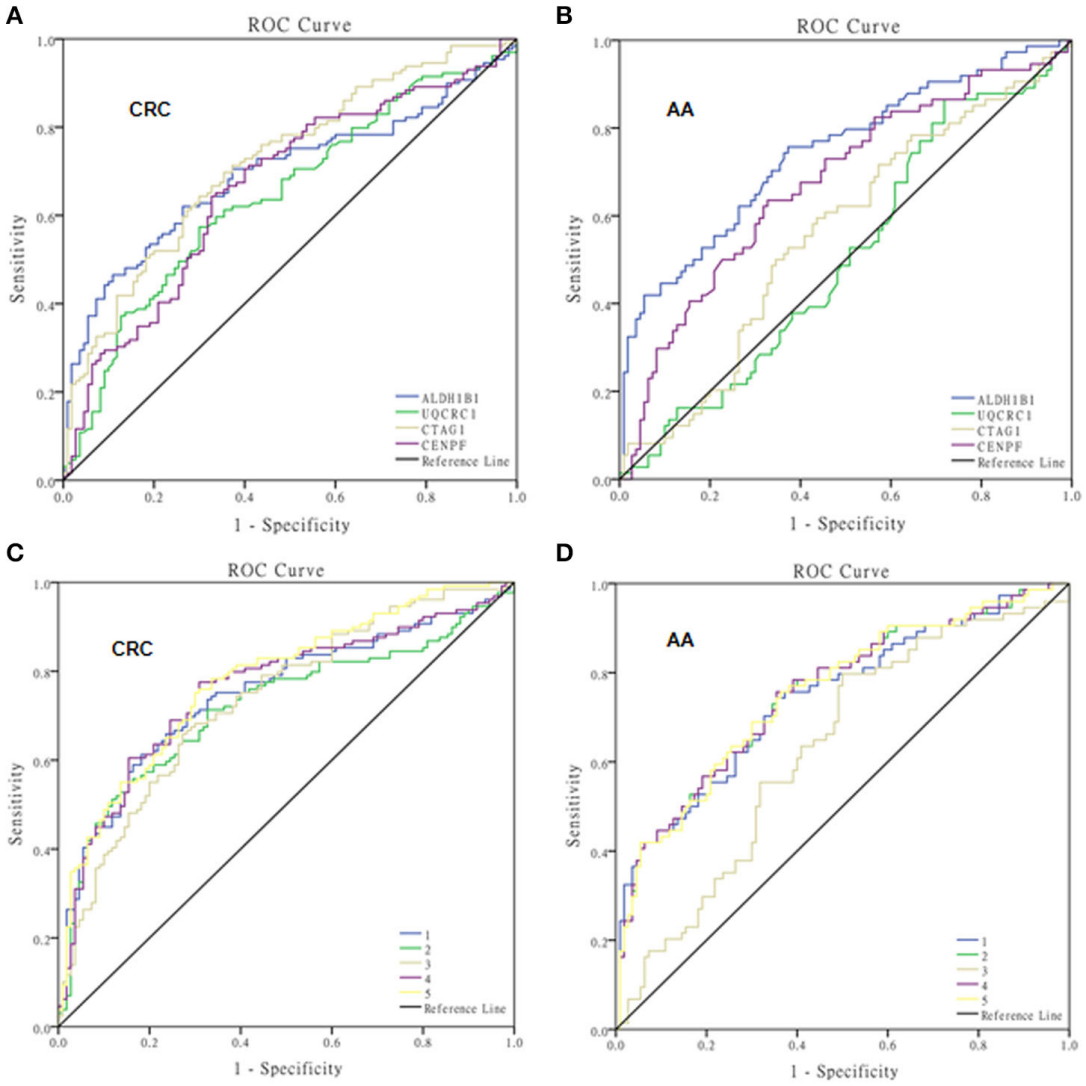
ROC curves of autoantibodies against selected TAAs examined by ELISAs. ROC curves of single TAAs of ALDH1B1, UQCRC1, CTAG1, and CENPF for colorectal cancer **(A)** and advanced adenoma **(B)**, and multiple panels of TAAs for colorectal cancer **(C)** and advanced adenoma **(D)**. 1, ALDH1B1+CTAG1; 2, ALDH1B1+CENPF; 3, CTAG1+CENPF; 4, ALDH1B1+CTAG1+CENPF; 5, ALDH1B1+CTAG1+CENPF+UQCRC1.

The test samples were the same as those used in the protein microarray. The levels of autoantibodies in AA and CRC samples compared with healthy control samples are presented in Figure 2. We constructed ROC curves and calculated the AUC for each autoantibody (Figure 3). The results for sensitivity, specificity, and *p*-value are shown in Table 1. When comparing healthy control and AA samples, the adjusted AUC of ALDH1B1 autoantibodies was 0.74 (95% CI: 0.66–0.82) with sensitivity of 75.68% and specificity of 63.06%. For CRC, the adjusted AUC was 0.70 (95% CI: 0.63–0.77) with sensitivity of 62.31% and specificity of 73.87%. Autoantibodies against CTAG1 also showed a high adjusted AUC (0.72, 95% CI: 0.65–0.79) in discriminating healthy control and CRC samples. The adjusted AUC of CENPF autoantibodies was 0.67 and 0.70 in discriminating healthy control and AA/CRC samples, respectively. When using a combination of all TAAbs, the adjusted AUC was 0.79 (95% CI: 0.71–0.85) in discriminating CRC patients, and the adjusted AUC was 0.79 (95% CI: 0.69–0.87) with sensitivity of 75.68% and specificity of 63.64% for discrimination of AA patients (Figure 3, Table 2).

**Table 1:** Detection performance of single tumor-associated autoantibodies in the detection of colorectal cancer and advanced adenoma determined by ELISA.

**Candidate TAAbs**	**AUC value (95% CI)**		**Adjusted AUC value (95% CI)**		**SE (%)**		**SP (%)**		***P*****-value**	
	**CRC**	**AA**	**CRC**	**AA**	**CRC**	**AA**	**CRC**	**AA**	**CRC**	**AA**
ALDH1B1	0.70 (0.63–0.76)	0.75 (0.67–0.82)	0.70 (0.63–0.77)	0.74 (0.66–0.82)	62.31	75.68	73.87	63.06	<0.001	<0.001
UQCRC1	0.65 (0.58–0.72)	0.51 (0.42–0.59)	0.63 (0.56–0.69)	0.65 (0.57–0.72)	57.70	86.49	70.27	27.93	<0.001	0.850
CTAG1	0.72 (0.66–0.79)	0.57 (0.48–0.64)	0.72 (0.65–0.79)	0.62 (0.55–0.68)	64.62	59.46	70.27	56.36	<0.001	0.169
CENPF	0.67 (0.60–0.74)	0.67 (0.59–0.75)	0.67 (0.61–0.74)	0.70 (0.63–0.77)	64.34	62.67	67.27	67.27	<0.001	<0.001

*ELISA, enzyme-linked immunosorbent assay; AUC, area under the curve; SE, sensitivity; SP, specificity; CI, 95% confidence interval; CRC, colorectal cancer; AA, advanced adenoma*.

**Table 2:** Detection performance of multiple tumor-associated autoantibodies determined by ELISA.

**Panel no**.	**AUC value (95% CI)**		**Adjusted AUC value (95% CI)**		**SE (%)**		**SP (%)**	
	**CRC**	**AA**	**CRC**	**AA**	**CRC**	**AA**	**CRC**	**AA**
1	0.75 (0.69–0.81)	0.74 (0.67–0.82)	0.75 (0.68–0.81)	0.74 (0.66–0.82)	61.24	74.32	18.12	64.55
2	0.72 (0.66–0.79)	0.75 (0.68–0.83)	0.72 (0.63–0.79)	0.76 (0.67–0.85)	55.04	75.68	84.55	63.64
3	0.74 (0.67–0.80)	0.63 (0.55–0.71)	0.74 (0.66–0.80)	0.67 (0.57–0.77)	65.89	79.73	72.73	50.00
4	0.76 (0.70–0.82)	0.75 (0.68–0.83)	0.76 (0.68–0.83)	0.76 (0.66–0.85)	77.52	75.68	69.09	64.55
5	0.78 (0.72–0.84)	0.75 (0.68–0.83)	0.79 (0.71–0.85)	0.79 (0.69–0.87)	75.19	75.68	70.00	63.64

*ELISA, enzyme-linked immunosorbent assay; AUC, area under the curve; SE, sensitivity; SP, specificity; CI, 95% confidence interval; CRC, colorectal cancer; AA, advanced adenoma; 1, ALDH1B1+CTAG1; 2, ALDH1B1+CENPF; 3, CTAG1+CENPF; 4, ALDH1B1+CTAG1+CENPF; 5, ALDH1B1+CTAG1+CENPF+UQCRC*.

**Figure 4:**
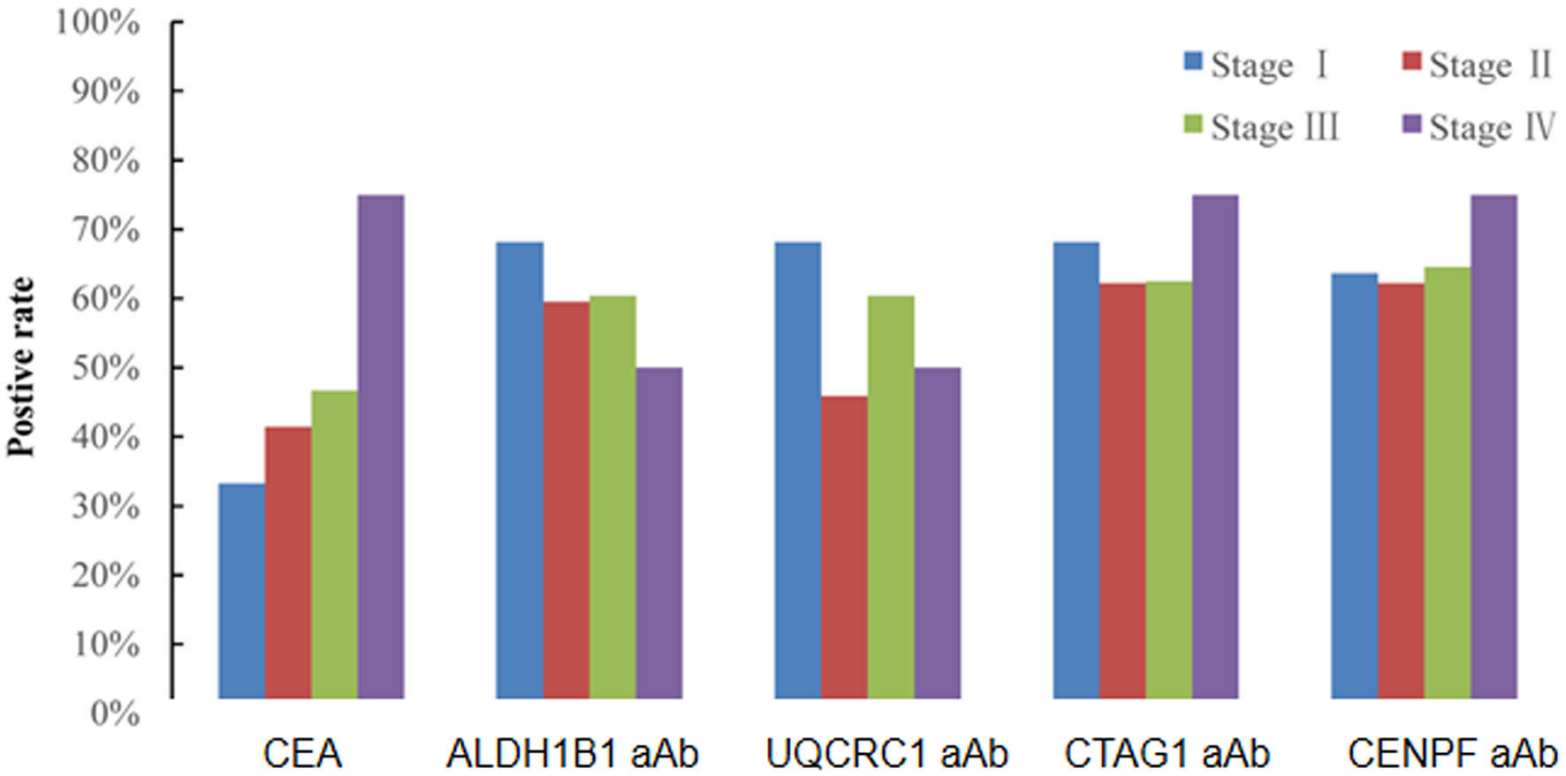
Comparison of the positive rates of CEA and autoantibodies against ALDH1B1, UQCRC1, CTAG1, and CENPF in CRC of different UICC stages.

**Figure 5:**
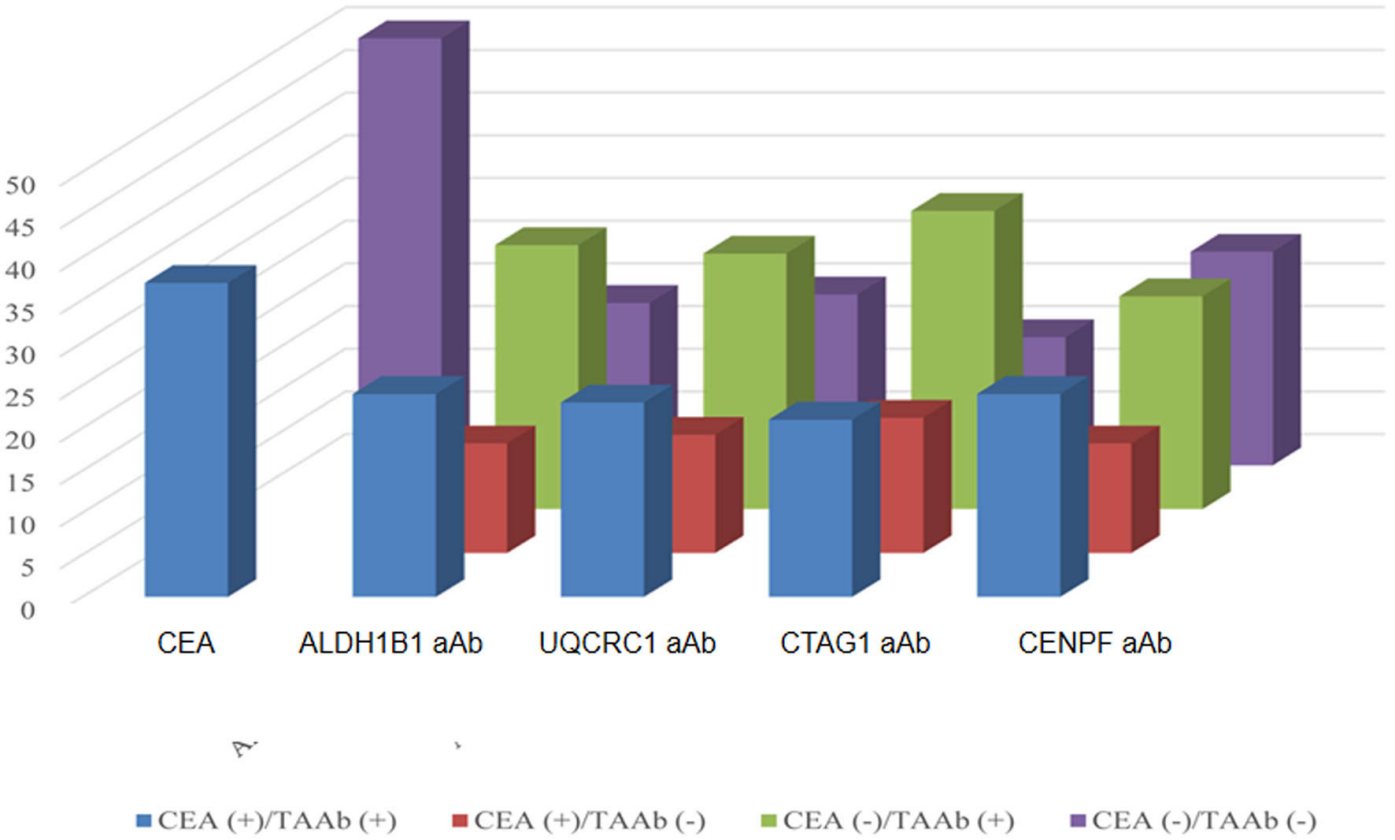
Comparison of the positivity rates of CEA and autoantibodies against TAAs in CRC patients. The numbers of positive and negative cases of autoantibodies against ALDH1B1, UQCRC1, CTAG1, and CENPF in CRC patients who were positive (in blue) or negative (in purple) for CEA.

Of the 130 CRC patients, 87 had available test results of CEA which were obtained from clinical records after their diagnosis during their treatment in the designated hospitals by the CRC screening program and 37 (42.5%) were positive (cutoff value of 5 ng/ml). Based on the cutoff value determined by the Youden index, the positive rates of autoantibodies against ALDH1B1, UQCRC1, CTAG1, and CENPF were 62.3, 57.7, 64.6, and 64.6%, respectively (Table S5). Specifically, for early-stage CRC, the positive rate of CEA was 38.6%, which was less than that of autoantibodies against ALDH1B1 (62.7%), UQCRC1 (54.2%), CTAG1 (64.4%), and CENPF (62.7%) (Figure 4). In CEA-negative CRCs, autoantibodies against ALDH1B1 (62.0%), UQCRC1 (60%), CTAG1 (70.0%), and CENPF (50.0%) were still useful to detect positive cases (Figure 5, Table 3).

**Table 3:** Autoantibodies positive for candidate tumor-associated antigens in colorectal cancer patients negative for CEA.

		**CEA**	**ALDH1B1 aAb**	**UQCRC1 aAb**	**CTAG1 aAb**	**CENPF aAb**
CEA (+)	TAAb (+)	37 (42.5%)	24 (64.9%)	23 (62.2%)	21 (56.8%)	24 (64.9%)
	TAAb (–)		13 (35.1%)	14 (37.8%)	16 (43.2%)	13 (35.1%)
CEA (–)	TAAb (+)	50 (57.5%)	31 (62.0%)	30 (60.0%)	35 (70.0%)	25 (50.0%)
	TAAb (–)		19 (38.0%)	20 (40.0%)	15 (30.0%)	25 (50%)

*aAb, autoantibody; CEA, carcinoembryonic antigen*.

### Western Blotting Confirms Higher Levels of ALDH1B1 Autoantibodies in Sera From AA and CRC Patients Than in That From Healthy Controls

**Figure 6:**
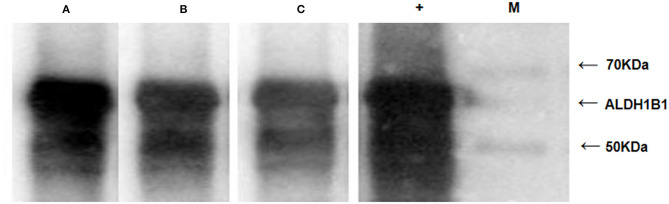
Western blots with recombinant ALDH1B1 protein to detect the serum level of ALDH1B1 autoantibodies. Serum samples were obtained from AA patients **(A)**, CRC patients **(B)**, and healthy controls **(C)**. The lane with “+” indicates an antibody against ALDH1B1 used as a positive control; M. EasySee Western Marker (TransGen Biotech, Beijing).

Western blotting was conducted with the 61.9-kDa recombinant ALDH1B1 protein (Figure S2) to examine the level of autoantibodies against ALDH1B1 in sera from AA/CRC patients and healthy controls. Sera from 30 CRC cases, 30 AA cases, and 30 healthy controls, which were used in previous ELISAs, were selected randomly, mixed, and used as the primary antibodies for western blot analysis. The results showed that the levels of autoantibodies against ALDH1B1 were higher in samples from CRC and AA patients, especially AA, compared with those from healthy controls (Figure 6), which was consistent with the ELISA results, in which the AUC values to discriminate AA and CRC were 0.75 and 0.70, respectively.

## Discussion

According to Poletaev et al. (11) the immune system can detect pathological changes from the earliest stages of a disease and respond to them by changing autoantibody production. Although the exact mechanisms responsible for cancer-related autoantibody production are still largely unknown, several theories have been proposed: (1) defects in tolerance and inflammation, (2) changes in protein expression levels, (3) altered protein structures, and (4) cellular death mechanisms (22). The theories have proven that the production of autoantibodies may be an early event in the transition to pre-cancer.

To discover novel autoantibodies, methods that allow simultaneous screening of multiple autoantibodies are required, which include recombinant cDNA expression cloning (SEREX), phage display, SERPA, multiple affinity protein profiling (MAPPing), and protein microarrays (23). ELISAs have always been the most commonly used method and are often used to confirm the detection value of autoantibodies screened by high-throughput methods. SERPA is very useful to identify novel TAAs with total proteins extracted from tumor tissue as an antigen library. Protein microarrays can screen a large number of TAAbs simultaneously and require little serum. In our study, we took full advantage of the superiority of SERPA, protein microarrays, and ELISAs to screen and confirm TAAbs with detection value.

In our SERPA analysis, CSRP1, SELENBP1, ALDH1B1, UQCRC1, and ENO1 showed differential immunological reactions between CRC patients and healthy controls. The CSRP1 gene is downregulated and might be involved in the progression of CRC (17). Downregulation of SELENBP1 expression is reactivated by inducing CRC differentiation (18). ALDH1B1 is crucial for colon tumorigenesis by modulating Wnt/β-Catenin, Notch, and PI3K/Akt signaling pathways (19). Downregulation of UQCRC1 has been correlated to lymph node metastasis and a poor prognosis of CRC (20). ENO1 was also reported to be associated with colorectal cancer (21). The abnormally regulated protein expression of the above genes in CRC implies the generation of corresponding autoantibodies, which may be potential CRC-related TAAs.

In the present study, protein microarray assays were conducted for high-throughput screening and comparison of the performance of 26 TAAs identified by our SERPA analysis, which have been reported in previous studies. Chen et al. (16) assessed 64 serum autoantibodies measured by multiplex bead-based serological assays but did not perform further verification. Although autoantibodies against IGF2BP1, CTAG1, ANXA4, RPH3AL, RPL13, and VIL1 have been reported in other studies (24–28), the results were variable in the various studies because some had small samples or different methods and judgment standards. Autoantibodies against HSP60, ACY1, PRDX3, RGN, HSP60, CENPF, AIF, HINT1, CALR, HMGN3, and MPHOSPH6 in CRC were examined for the first time. In the present study, we further evaluated and compared the detection potentials of these TAAbs by microarray analysis. The results showed that autoantibodies against CENPF and CTAG1 may have underlying detection values for CRC/AA, and ALDH1B1, UQCRC1, IMPDH2, MDM2, HSP60, and RPL13 for CRC.

ELISA is considered to be a more accurate method to detect an antigen and autoantibodies. In the present study, autoantibodies against ALDH1B1, UQCRC1, CTAG1, and CENPF were validated by ELISAs and the results confirmed their detection value for CRC/AA. Specifically, ALDH1B1 was powerful in detecting AA, which was confirmed by a western blot assay, whereas CTAG1 had a detection value to recognize CRC. This is the first study to demonstrate the detection value of ALDH1B1 for CRC and AA. When a combination of ALDH1B1, UQCRC1, CTAG1, and CENPF autoantibodies was used, an adjusted AUC value of 0.79 was reached for the detection of both CRC and AA.

In the European Group on Tumor Markers (EGTM) guidelines for the use of biomarkers in gastrointestinal cancer to screen colorectal cancer, preoperative CEA levels may be combined with clinical and histopathological criteria to determine the prognosis of patients with newly diagnosed CRC. However, CEA is not considered as a good biomarker for CRC screening because of its inadequate specificity and sensitivity. In the present study, we found higher positive rates of autoantibodies against ALDH1B1, UQCRC1, CTAG1, and CENPF with positive rates of 62.7, 54.2, 64.4, and 62.7%, respectively, in early CRC compared with that of 38.6% for CEA, and more than half of the early CRC cases negative for CEA were positive for these TAAbs, implying the potential value of these TAAbs for screening and early detection of CRC.

Although nearly 60% of examined individual autoantibody markers have shown relatively low sensitivity (<25%), good diagnostic performance (sensitivity: >60%; specificity: >80%) has been reported for three individual markers (SPAG9, RPH3AL, and CCDC83) (16). By comparison with these three markers, the autoantibody markers identified in the present study did not have higher sensitivity or specificity. However, high-throughput analysis was conducted in the present study to evaluate and compare 26 potential autoantibody markers to detect CRC, in which several novel autoantibody biomarkers were identified and validated. In addition, our samples included a large number of AA cases, and we compared their diagnostic value in AA with CRC and healthy groups.

Fecal immunochemical test (FIT) is the most widely used non-invasive test for CRC screening. Although the FIT has many benefits for CRC screening, it also has several limitations such as poor participation and low sensitivity to detect advanced adenoma. In such circumstances, many efforts have been made to search for suitable biomarkers for early detection of CRC. A blood test might be an ideal candidate considering its ease of use that could potentially improve participation and compliance. Although the diagnostic performance for detecting CRC (sensitivity >60%; specificity >80%) of the three individual markers SPAG9, RPH3AL, and CCDC83 was inferior to that for FIT, the panel of the autoantibodies had higher sensitivity for detecting AA than FIT. The blood-based biomarker therefore might be a complementary test as for the current screening modalities, which deserves further validation in other populations.

The generation of autoantibodies against TAAs is not fully understood. TAA proteins are most likely either mutated, overexpressed, posttranslationally modified, misfolded, cleaved aberrantly, or localized aberrantly in tumor cells (12). In the present study, we revealed the promising detection value of autoantibodies against ALDH1B1 in CRC and AA, especially AA. ALDH1B1 is an important isoform in the ALDH1 family, which contributes to ALDH1 activity, and is presumed to promote the differentiation of stem cells and may be crucial for colon tumorigenesis (19). ALDH1B1 has been reported to be highly expressed in CRC and advanced colorectal adenoma, (29, 30) implying a possible mechanism for the generation of autoantibodies in CRC and AA. However, the exact mechanisms require further study.

There are several limitations of our study when interpreting the results. First, although the samples were selected from a large-scale population-based CRC screening program in China, we only included a limited number of samples; therefore, we cannot rule out the probability of selection bias. However, we anticipated to validate the significant findings of this study in larger samples in the future. Second, although we identified a series of autoantibodies with potent detection values, we did not examine them all by ELISAs. Third, the laboratory test results of CEA were obtained from clinical records for some patients after their diagnosis of CRC and missing data existed for some participants, which may therefore lead to biased interpretation of the comparison of the diagnostic performance between the CEA and the autoantibodies, which should be further validated in other studies. Fourth, we did not validate the TAAbs in an independent cohort, even though we conducted a state-of-the-art technique (bootstrapping) to minimize potential overfitting of the constructed multi-marker algorithms. We will study the contributions of the above factors and evaluate the detection values of the TAAs in another independent cohort in the future.

In summary, high-throughput screening by SERPA, protein microarray, and validation by ELISA indicated that ALDH1B1 autoantibodies have potential detection values for AA and CRC, and measuring serum autoantibodies against TAAs may improve the detection of early CRC.

## Data Availability Statement

All datasets presented in this study are included in the article/Supplementary Material.

## Ethics Statement

The studies involving human participants were reviewed and approved by Clinical Research Ethics Committee of Beijing Friendship Hospital, Capital Medical University, and Clinical Research Ethics Committee of Cancer Hospital, Chinese Academy of Medical Sciences. The patients/participants provided their written informed consent to participate in this study.

## Author Contributions

JH, HC, and HW contributed to the project design. BZ, XZ, JW, ZB, BC, NL, MD, and HC participated in collecting samples. HW, BZ, XL, YL, SJ, and SQ participated in implementation of the experiments. JH, HC, AX, and DZ contributed significantly to drafting the manuscript, reviewing the literature, and revising the manuscript critically. All authors read and approved the final manuscript.

## Conflict of Interest

The authors declare that the research was conducted in the absence of any commercial or financial relationships that could be construed as a potential conflict of interest.

## Supplementary Material

Figure S1 Representative results of SERPA analysis of autoantibodies in sera from CRC patients and normal serum. **(A)** Coomassie blue-stained 2-DE of proteins isolated from CRC tissue lysates. Arrows in the 2D gel indicate the most immunoreactive spots recognized by serum from CRC patients. **(B)** CRC patient and **(C)** normal control sera.

Figure S2 SDS-PAGE to prepare the recombinant ALDH1B1 protein. 1, DNA marker; 2, uninduced lysate; 3, induced lysate; 4, supernatant of the lysate after ultrasonication; 5, precipitation of the lysate after ultrasonication; 6, flow through after loading; 7, flow through after binding; 8, eluted recombinant ALDH1B1 protein.

Table S1 Clinical characteristics of research subjects.

Table S2 Candidatetumour-associated autoantigens applied for microarray analysis identified by serological proteome analysis and MALDI-TOF mass spectrometry.

Table S3 Information about antigen proteins for the preparation of protein microarray.

Table S4 Diagnostic performance of 26 single tumor-associated autoantibodies in the detection of colorectal cancer and advanced adenoma.

Table S5 Comparison of seroprevalence between the autoantibodies and CEA in colorectal cancer at various stages.
