# Supplementary figures and images for: Identification and Validation of Novel Serum Autoantibody Biomarkers for Early Detection of Colorectal Cancer and Advanced Adenoma
Source: Front Oncol. 2020 Jul 22;10:1081. doi: 10.3389/fonc.2020.01081 (PMC7387658; doi:10.3389/fonc.2020.01081)

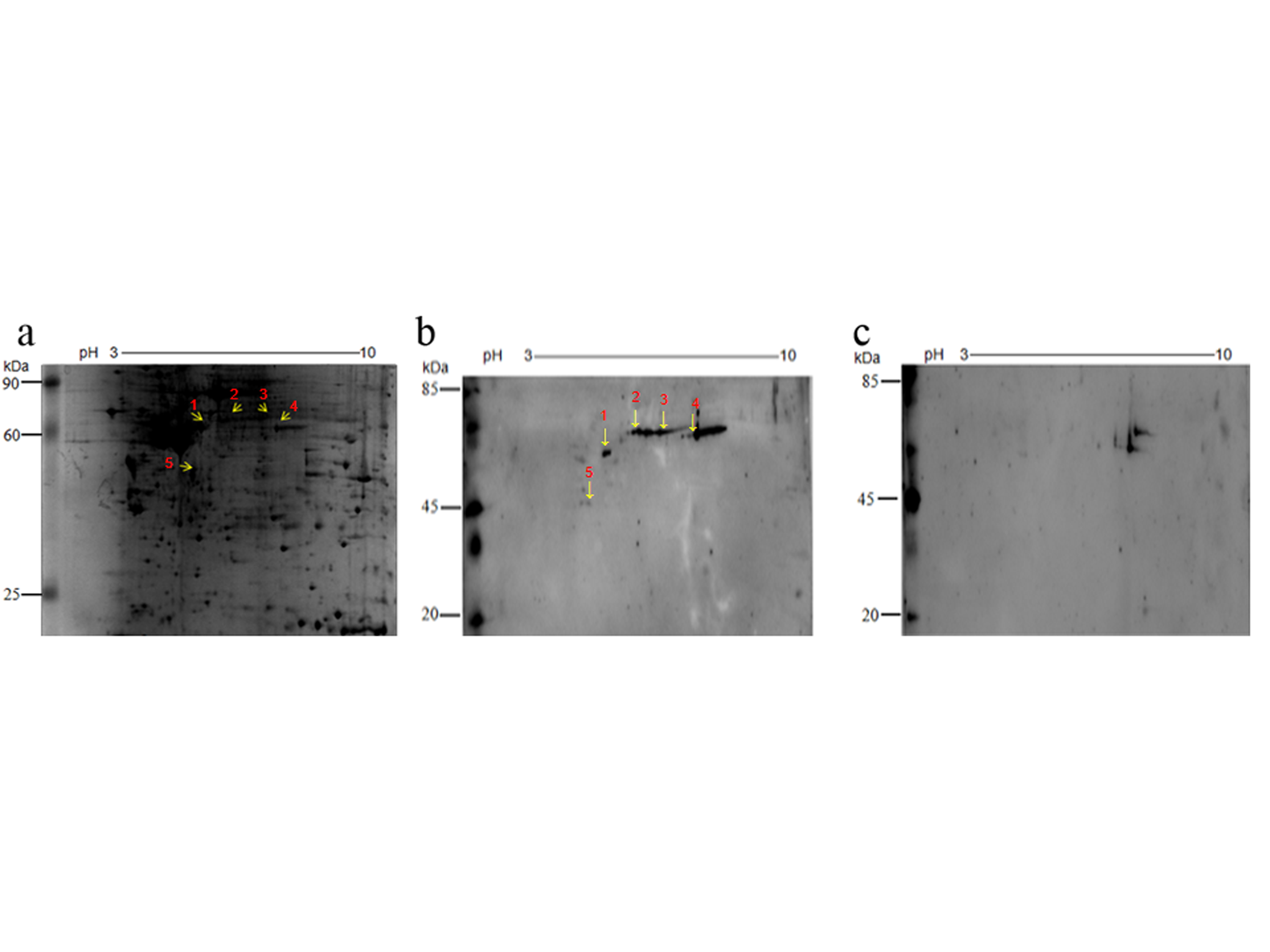

Supplement: Figure S1 — Representative results of SERPA analysis of autoantibodies in sera from CRC patients and normal serum. (A) Coomassie blue-stained 2-DE of proteins isolated from CRC tissue lysates. Arrows in the 2D gel indicate the most immunoreactive spots recognized by serum from CRC patients. (B) CRC patient and (C) normal control sera. [file Image_1.TIF]

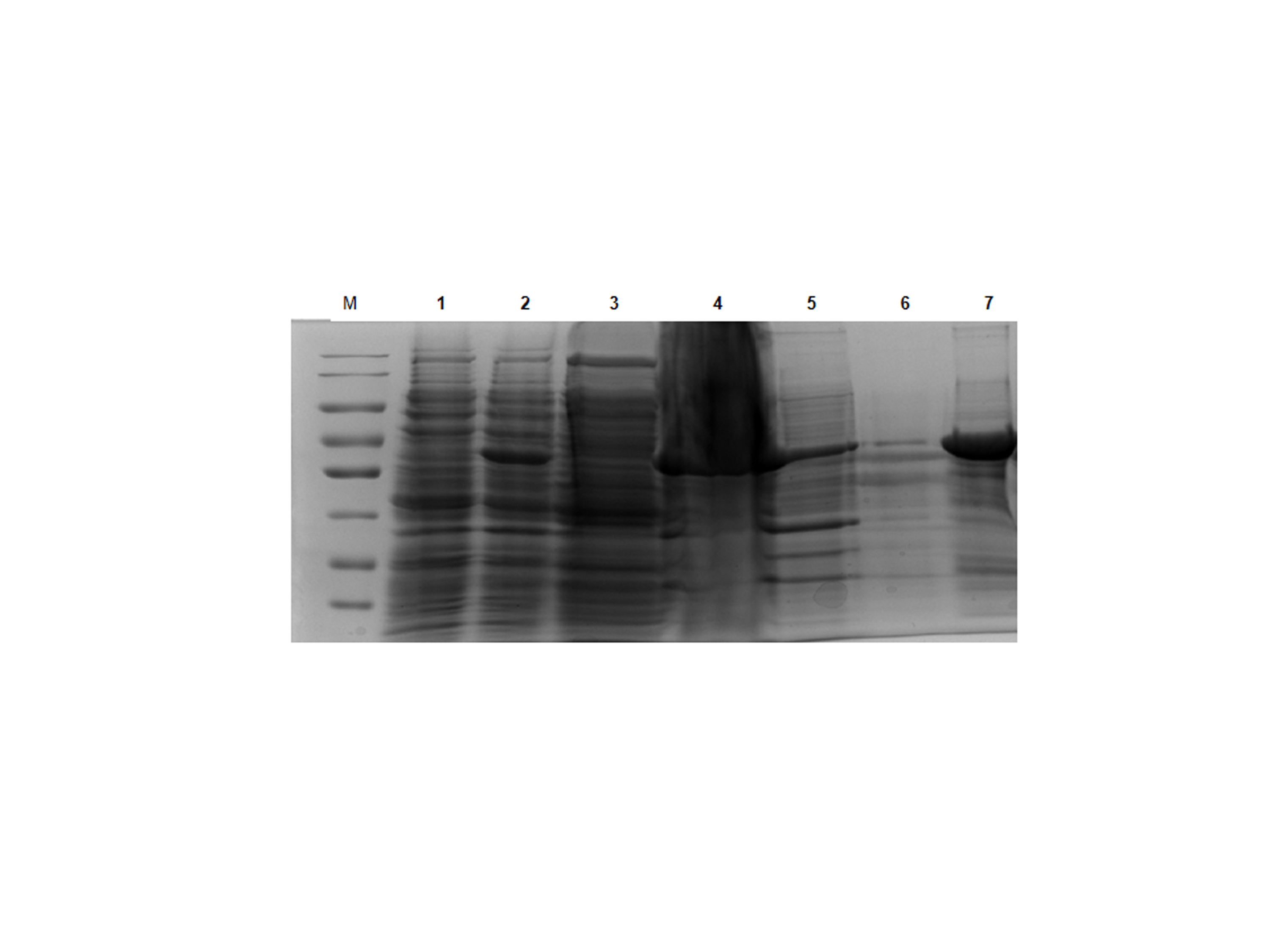

Supplement: Figure S2 — SDS-PAGE to prepare the recombinant ALDH1B1 protein. 1, DNA marker; 2, uninduced lysate; 3, induced lysate; 4, supernatant of the lysate after ultrasonication; 5, precipitation of the lysate after ultrasonication; 6, flow through after loading; 7, flow through after binding; 8, eluted recombinant ALDH1B1 protein. [file Image_2.TIF]
