# Supplementary material for: Identification and Validation of Novel Serum Autoantibody Biomarkers for Early Detection of Colorectal Cancer and Advanced Adenoma
Source: Front Oncol. 2020 Jul 22;10:1081. doi: 10.3389/fonc.2020.01081 (PMC7387658; doi:10.3389/fonc.2020.01081)
Supplement: Table S1 — Clinical characteristics of research subjects. [file Table_1.DOCX]

Table S1. Clinical characteristics of research subjects

| Variables | Numbers | Age mean | Median (range) | Gender (male/female) |
| --- | --- | --- | --- | --- |
| Colorectal cancer | 130 | 56 | 57 (22-80)^a^ | 80/50^c^ |
| UICC stageⅠ | 22 | 61 | 61 (42-80) | 16/6 |
| Ⅱ | 37 | 56 | 58 (38-77) | 24/13 |
| Ⅲ | 48 | 55 | 55 (29-80) | 29/19 |
| Ⅳ | 12 | 56 | 55 (35-74) | 6/6 |
| Missing | 11 | 53 | 57 (22-66) | 5/6 |
| Advanced adenoma | 75 | 59 | 60 (41-70)^b^ | 43/32^d^ |
| Healthy control | 110 | 59 | 60 (41-71) | 65/45 |

^a^ p = 0.051; ^b^ p = 0.596; ^c^ p = 0.699; ^d^ p = 0.812
