# Supplementary material for: Identification and Validation of Novel Serum Autoantibody Biomarkers for Early Detection of Colorectal Cancer and Advanced Adenoma
Source: Front Oncol. 2020 Jul 22;10:1081. doi: 10.3389/fonc.2020.01081 (PMC7387658; doi:10.3389/fonc.2020.01081)
Supplement: Table S2 — Candidatetumour-associated autoantigens applied for microarray analysis identified by serological proteome analysis and MALDI-TOF mass spectrometry. [file Table_2.DOCX]

**Table S2.** Candidate tumour-associated autoantigens applied for microarray analysis identified by serological proteome analysis and MALDI-TOF mass spectrometry

| Spot no. | Protein | Mass | MW/PI | Sequence coverage | Match peptides | Score | Function |
| --- | --- | --- | --- | --- | --- | --- | --- |
| 1 | UQCRC1 | 53297 | 52646/5.94 | 53% | 15 | 193 | Proliferation |
| 3 | SELENBP1 | 52928 | 52391/5.93 | 47% | 18 | 267 | Transport |
| 3 | ALDH1B1 | 57658 | 57238/6.36 | 24% | 12 | 131 | Metabolism |
| 4 | ENO1 | 47481 | 47169/7.01 | 44% | 18 | 598 | Metabolism |
| 5 | CSRP1 | 21409 | 20567/8.90 | 59% | 10 | 617 | Cell signal |

MALDI-TOF, matrix-assisted laser desorption/ionization-time-of-flight; MW, molecular weight; PI, isoelectric point.
