# Supplementary material for: Identification and Validation of Novel Serum Autoantibody Biomarkers for Early Detection of Colorectal Cancer and Advanced Adenoma
Source: Front Oncol. 2020 Jul 22;10:1081. doi: 10.3389/fonc.2020.01081 (PMC7387658; doi:10.3389/fonc.2020.01081)
Supplement: Table S3 — Information about antigen proteins for the preparation of protein microarray. [file Table_3.DOCX]

**Table S3.** Information about antigen proteins for the preparation of protein microarray

| Abbrevation | Antigen protein | Protein sequence | Original Concentration (ug/ul) | Spotting concentration (ug/ul) | Manufacturer |
| --- | --- | --- | --- | --- | --- |
| TP53 | Cellular tumor antigen p53 | 1-393 | 0.06 | 0.02 | Abnova |
| IMPDH2 | Inosine-5'-monophosphate dehydrogenase 2 | 1-514 | 0.16 | 0.80 | Abnova |
| MAGEA4 | Melanoma-associated antigen 4 | 1-317 | 0.19 | 0.95 | Abnova |
| MDM2 | E3 ubiquitin-protein ligase Mdm2 | 1-491 | 0.06 | 0.30 | Abnova |
| ALDH1B1 | Aldehyde dehydrogenase 1 family member B1 | 1-517 | 0.08 | 0.40 | Abnova |
| UQCRC1 | Ubiquinol-cytochrome c reductase core protein 1 | 1-480 | 0.07 | 0.35 | Abnova |
| RPL13 | 60S ribosomal protein L13 | 1-211 | 0.02 | 0.10 | Abnova |
| RPH3AL | Rab effector Noc2 | 20-91 | 3.60 | 0.36 | Novus |
| HMGN3 | High mobility group nucleosome-binding domain-containing protein 3 | 1-77 | 0.19 | 0.06 | Abnova |
| MPHOSPH6 | M-phase phosphoprotein 6 | 1-160 | 0.21 | 0.21 | Abnova |
| IGF2BP1 | Insulin-like growth factor 2 mRNA-binding protein 1 | 1-577 | 0.03 | 0.03 | Abnova |
| VIL1 | Villin 1 | 1-421 | 0.05 | 0.05 | Abnova |
| ENO1 | Enolase 1 | 1-434 | 0.13 | 0.26 | Abnova |
| HSP60 | Heat shock protein 60 | 1-573 | 0.30 | 0.15 | Abcam |
| CENPF | Centromere protein F | 121-220 | 1.20 | 0.4 | Abnova |
| RGN | Regucalcin | 1-299 | 0.06 | 0.06 | Abnova |
| PRDX3 | peroxiredoxin 3 | 1-256 | 1.00 | 1.0 | Abnova |
| ACY1 | Aminoacylase-1 | 1-408 | 0.05 | 0.025 | Abnova |
| ANXA4 | Annexin A4 | 1-321 | 0.19 | 0.19 | Abnova |
| SELENBP1 | Selenium binding protein 1 | 1-472 | 0.09 | 0.09 | Abnova |
| CSRP1 | Cysteine and glycine rich protein 1 | 1-193 | 0.11 | 0.22 | Abnova |
| AIF1 | Apoptosis inducing factor 1 | 1-147 | 0.95 | 0.95 | Abnova |
| HINT1 | Histidine triad nucleotide binding protein 1 | 1-126 | 1.0 | 1.0 | Novus |
| CTAG1 | Cancer/testis antigen 1B | 1-180 | 0.68 | 1.4 | House made |
| MYH13 | Myosin heavy chain 13 | 250-462, 760-992 | 8.04 | 4.02 | House made |
| CALR | Calreticulin | 1-417 | 0.11 | 0.11 | Abnova |
