# Supplementary material for: Identification and Validation of Novel Serum Autoantibody Biomarkers for Early Detection of Colorectal Cancer and Advanced Adenoma
Source: Front Oncol. 2020 Jul 22;10:1081. doi: 10.3389/fonc.2020.01081 (PMC7387658; doi:10.3389/fonc.2020.01081)
Supplement: Table S4 — Diagnostic performance of 26 single tumor-associated autoantibodies in the detection of colorectal cancer and advanced adenoma. [file Table_4.DOCX]

**Table S4.** Diagnostic performance of 26 single tumor-associated autoantibodies in the detection of colorectal cancer and advanced adenoma.

| TAAbs | AUC value | | 95% CI | | SE (%) | | | SP (%) | | P-Value | |
| --- | --- | --- | --- | --- | --- | --- | --- | --- | --- | --- | --- |
|  | CRC | AA | CRC | AA | | CRC | AA | CRC | AA | CRC | AA |
| **ALDH1B1*** | 0.62 | 0.51 | 0.55-0.70 | 0.42-0.60 | | 37.60 | 36.23 | 84.68 | 72.97 | 0.001 | 0.823 |
| **UQCRC1*** | 0.64 | 0.51 | 0.57-0.71 | 0.42-0.60 | | 47.20 | 49.28 | 78.38 | 63.96 | <0.001 | 0.880 |
| **SELENBP1** | 0.58 | 0.50 | 0.51-0.65 | 0.41-0.60 | | 54.40 | 47.83 | 65.77 | 59.46 | 0.033 | 0.966 |
| **CSRP1** | 0.57 | 0.52 | 0.50-0.64 | 0.43-0.61 | | 59.20 | 60.87 | 55.86 | 51.35 | 0.071 | 0.698 |
| **ENO1** | 0.57 | 0.56 | 0.50-0.64 | 0.47-0.65 | | 69.60 | 30.43 | 47.75 | 90.09 | 0.069 | 0.197 |
| CENPF* | 0.58 | 0.64 | 0.51-0.65 | 0.55-0.72 | | 36.00 | 59.42 | 80.18 | 69.37 | 0.035 | 0.0020 |
| CTAG1* | 0.55 | 0.59 | 0.47-0.62 | 0.50-0.68 | | 49.60 | 57.97 | 63.06 | 64.86 | 0.207 | 0.041 |
| TP53 | 0.67 | 0.65 | 0.60-0.74 | 0.57-0.73 | | 48.80 | 75.36 | 76.58 | 55.86 | <0.001 | 0.001 |
| IMPDH2 | 0.62 | 0.50 | 0.54-0.69 | 0.41-0.59 | | 71.20 | 18.84 | 48.65 | 96.40 | 0.002 | 0.947 |
| MAGEA4 | 0.57 | 0.51 | 0.50-0.64 | 0.42-0.60 | | 32.00 | 55.07 | 81.08 | 54.95 | 0.061 | 0.873 |
| MDM2 | 0.62 | 0.51 | 0.55-0.70 | 0.42-0.61 | | 48.00 | 23.19 | 78.38 | 93.69 | 0.001 | 0.755 |
| RPL13 | 0.63 | 0.51 | 0.56-0.70 | 0.42-0.60 | | 57.60 | 42.03 | 65.77 | 70.27 | <0.001 | 0.903 |
| RPH3AL | 0.52 | 0.51 | 0.45-0.59 | 0.49-0.66 | | 24.00 | 72.46 | 88.29 | 45.05 | 0.603 | 0.113 |
| HMGN3 | 0.51 | 0.57 | 0.43-0.58 | 0.49-0.66 | | 79.20 | 65.22 | 30.63 | 51.35 | 0.900 | 0.102 |
| MPHOSPH6 | 0.54 | 0.55 | 0.47-0.62 | 0.47-0.64 | | 68.80 | 85.51 | 43.24 | 29.73 | 0.266 | 0.221 |
| IGF2BP1 | 0.54 | 0.54 | 0.47-0.61 | 0.45-0.63 | | 61.60 | 23.19 | 53.15 | 89.19 | 0.304 | 0.366 |
| VIL1 | 0.58 | 0.55 | 0.50-0.65 | 0.46-0.64 | | 64.80 | 84.06 | 55.86 | 27.03 | 0.048 | 0.260 |
| HSP60 | 0.60 | 0.59 | 0.53-0.67 | 0.50-0.67 | | 41.60 | 34.78 | 79.28 | 84.68 | 0.009 | 0.056 |
| RGN | 0.59 | 0.52 | 0.51-0.66 | 0.43-0.60 | | 52.80 | 15.94 | 69.37 | 97.30 | 0.024 | 0.743 |
| PRDX3 | 0.59 | 0.53 | 0.52-0.67 | 0.44-0.62 | | 48.80 | 37.68 | 74.77 | 73.87 | 0.014 | 0.544 |
| ACY1 | 0.54 | 0.56 | 0.46-0.61 | 0.48-0.65 | | 17.60 | 60.87 | 91.89 | 51.35 | 0.324 | 0.158 |
| ANXA4 | 0.59 | 0.54 | 0.52-0.67 | 0.45-0.63 | | 40.80 | 43.48 | 80.18 | 70.27 | 0.013 | 0.359 |
| AIF1 | 0.50 | 0.54 | 0.43-0.58 | 0.45-0.63 | | 64.00 | 69.57 | 45.05 | 43.24 | 0.919 | 0.377 |
| HINT1 | 0.55 | 0.51 | 0.47-0.62 | 0.43-0.60 | | 53.60 | 24.64 | 58.56 | 82.88 | 0.210 | 0.752 |
| MYH13 | 0.50 | 0.50 | 0.43-0.58 | 0.41-0.59 | | 98.40 | 36.23 | 8.11 | 72.07 | 0.983 | 0.946 |
| CALR | 0.56 | 0.53 | 0.48-0.63 | 0.44-0.61 | | 52.80 | 59.42 | 58.56 | 54.95 | 0.143 | 0.571 |

AUC, area under curve; SE, sensitivity; SP, specificity; CI, 95% confidence interval; CRC, colorectal cancer; AA, advanced adenoma; Bold, screened by SERPA ; *, applied for ELISA assay
