# Supplementary material for: Identification and Validation of Novel Serum Autoantibody Biomarkers for Early Detection of Colorectal Cancer and Advanced Adenoma
Source: Front Oncol. 2020 Jul 22;10:1081. doi: 10.3389/fonc.2020.01081 (PMC7387658; doi:10.3389/fonc.2020.01081)
Supplement: Table S5 — Comparison of seroprevalence between the autoantibodies and CEA in colorectal cancer at various stages. [file Table_5.DOCX]

**Table S5. Comparison of seroprevalence between the autoantibodies and CEA in colorectal cancer at various stages**

| parameters | CEA | ALDH1B1 aAb | P-value | UQCRC1aAb | P-value | CTAG1 aAb | P-value | CENPF aAb | P-value |
| --- | --- | --- | --- | --- | --- | --- | --- | --- | --- |
| Colorectal cancer | 37/87  (42.5%) | 81/130  (62.3%) | 0.004 | 75/130 (57.7%) | 0.028 | 84/130 (64.6%) | 0.001 | 84/130  (64.6%) | 0.001 |
| UICC stage | 37/82  (45.1%) | 72/119  (60.5%) | 0.021 | 67/119 (56.3%) | 0.085 | 77/119 (64.7%) | 0.003 | 77/119  (64.7%) | 0.003 |
| UICC stage Ⅰ | 5/15  (33.3%) | 15/22  (68.2%) | 0.037 | 15/22  (68.2%) | 0.037 | 15/22 (68.2%) | 0.037 | 14/22  (63.6%) | 0.070 |
| Ⅱ | 12/29  (41.4%) | 22/37  (59.5%) | 0.145 | 17/37  (45.9%) | 0.711 | 23/37 (62.2%) | 0.093 | 23/37  (62.2%) | 0.093 |
| Ⅲ | 14/30  (46.7%) | 29/48  (60.4%) | 0.235 | 29/48  (60.4%) | 0.235 | 30/48 (62.5%) | 0.170 | 31/48  (64.6%) | 0.119 |
| Ⅳ | 6/8  (75.0%) | 6/12  (50.0%) | 0.373 | 6/12  (50.0%) | 0.373 | 9/12  (75.0%) | 1.000 | 9/12  (75.0%) | 1.000 |
| Early stages  (I + II) | 17/44  (38.6%) | 37/59  (62.7%) | 0.016 | 32/59  (54.2%) | 0.117 | 38/59 (64.4%) | 0.009 | 37/59  (62.7%) | 0.016 |
| Late stages  (III + IV) | 20/38  (52.6%) | 35/60  (58.3%) | 0.579 | 35/60  (58.3%) | 0.579 | 39/60  (65%) | 0.223 | 40/60  (66.7%) | 0.165 |

aAb: autoantibody;; CRC, colorectal cancer; CEA,carcinoembryonic antigen
